# Active Surveillance for Avian Influenza Virus, Egypt, 2010–2012

**DOI:** 10.3201/eid2004.131295

**Published:** 2014-04

**Authors:** Ghazi Kayali, Ahmed Kandeil, Rabeh El-Shesheny, Ahmed S. Kayed, Mokhtar M. Gomaa, Asmaa M. Maatouq, Mahmoud M. Shehata, Yassmin Moatasim, Ola Bagato, Zhipeng Cai, Adam Rubrum, Mohamed A. Kutkat, Pamela P. McKenzie, Robert G. Webster, Richard J. Webby, Mohamed A. Ali

**Affiliations:** St. Jude Children's Research Hospital, Memphis, Tennessee, USA (G. Kayali, A. Rubrum, P.P. McKenzie, R.G. Webster, R.J. Webby);; National Research Center, Giza, Egypt (A. Kandeil, R. El-Shesheny, A.S. Kayed, M.M. Gomaa, A.M. Maatouq, M.M. Shehata, Y. Moatasim, O. Bagato, M.A. Kutkat, M.A. Ali);; Georgia State University, Atlanta, Georgia, USA (Z. Cai)

**Keywords:** avian influenza, H5N1, surveillance, Egypt, viruses, highly pathogenic avian influenza, HPAI, poultry

## Abstract

Continuous circulation of influenza A(H5N1) virus among poultry in Egypt has created an epicenter in which the viruses evolve into newer subclades and continue to cause disease in humans. To detect influenza viruses in Egypt, since 2009 we have actively surveyed various regions and poultry production sectors. From August 2010 through January 2013, >11,000 swab samples were collected; 10% were positive by matrix gene reverse transcription PCR. During this period, subtype H9N2 viruses emerged, cocirculated with subtype H5N1 viruses, and frequently co-infected the same avian host. Genetic and antigenic analyses of viruses revealed that influenza A(H5N1) clade 2.2.1 viruses are dominant and that all subtype H9N2 viruses are G1-like. Cocirculation of different subtypes poses concern for potential reassortment. Avian influenza continues to threaten public and animal health in Egypt, and continuous surveillance for avian influenza virus is needed.

In 2008, highly pathogenic avian influenza (HPAI) A(H5N1) virus became enzootic among poultry in Egypt, and the country became an epicenter for virus activity (*1*). As the established viruses drifted over time, viral genetic and antigenic diversity was generated. During 2010–2011, subclade 2.2.1 viruses (direct-drift progeny of the initially introduced virus) and 2.2.1.1 viruses (which might have emerged because of vaccine pressure) were cocirculating among poultry in Egypt (*2*). These subclades differed genetically and antigenically, hence complicating control efforts, especially vaccination (*3*). Subclade 2.2.1 viruses, commonly isolated from backyard flocks that are not vaccinated, caused all of the human cases in Egypt; from 2006 through September 2013, the toll rose to 173 cases and 63 deaths (*4*,*5*). Subclade 2.2.1.1 viruses were more prevalent on commercial farms, where vaccines are more frequently used (*6*). Furthermore, recent reports have indicated that very few mutations are needed for subtype H5N1 to become transmissible among ferrets, the best mammalian model of human influenza infection (*7*,*8*). In Egypt, a subtype H5N1 virus was found to have 2 of the 4 mutations needed to gain the transmissibility function, thereby underlying the need and urgency for surveillance among poultry (*8*). The Nile Delta region of Egypt was also identified as an area where substantial reassortment of influenza viruses can take place (*9*). As a further complication, in 2011, subtype H9N2 viruses were detected in poultry from areas in Egypt where subtype H5N1 viruses circulate (*10*).

Since 2009, we have been conducting systematic, active surveillance of avian influenza virus (AIV) among poultry in Egypt; the same locations are sampled over time, regardless of whether a clinical outbreak of disease is present. We previously reported that the threat of HPAI (H5N1) virus is widespread beyond rural areas and that the commercial sector is a key reservoir for virus transmission (*11*). Here we provide an update on the changing epizootiology and genetic features of AIV in Egypt and report co-infection of poultry in Egypt with influenza virus subtypes H5N1 and H9N2.

## Materials and Methods

### Sample Collection and Processing

**Figure 1:**
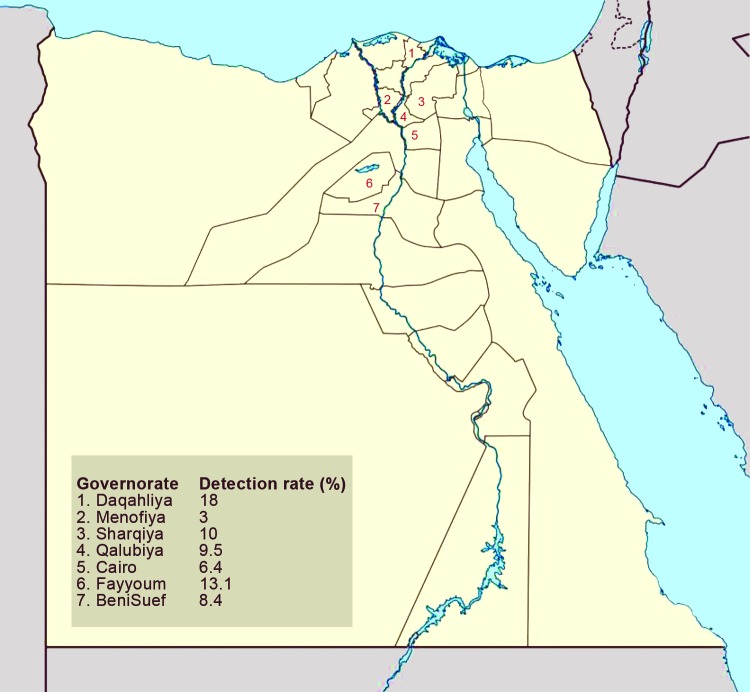
Location of surveillance governorates and percentage of avian influenza virus detection in each governorate, Egypt, 2010–2012.

A team of veterinarians collected cloacal and oropharyngeal swab samples from 11,452 birds from 4 poultry production sectors: commercial farms, backyard flocks, live-bird markets, and abattoirs. One swab sample was collected per bird, and depending on the size of the population, as many as 5 birds were sampled per flock. Birds were not randomly selected; samples were also collected from sick or dead birds found on site. From August 2010 through January 2013, a total of 6,904 cloacal and 4,548 oropharyngeal samples were collected from 63 sites in 7 governorates in Egypt, including Cairo (4 neighborhoods); 4 Nile Delta governorates (Qalubiya [12 villages], Menofiya [9 villages], Sharqiya [3 towns], and Daqahliya [4 towns]); and 2 mid-Egypt governorates (Fayyoum [22 villages] and BeniSuef [9 villages]) (Figure 1). The selected governorates represent the main foci of the poultry industry in Egypt and sites of previous AIV detection (*11*). The selected sampling sites were areas at which the veterinarian was known to the local population and thus had access to the poultry. The sites were routinely visited on a monthly basis regardless of the occurrence of clinical signs or poultry deaths. Study veterinarians subjectively recorded their field observations. Swab samples were collected in medium containing 50% glycerol, 50% phosphate-buffered saline (PBS), penicillin (2 × 10^6^ U/L), streptomycin (200 mg/L), and amphotericin B (250 mg/L) (antimicrobial drugs from Lonza, Walkersville, MD, USA). Samples were chilled on ice until delivered to the laboratory (within 24 hours). All samples were stored at –80°C until used.

### Screening for AIV 

For detection of AIV, 100 µL from 5 samples was pooled; RNA extracted from pools collected up to July 2012 was subjected to reverse transcription PCR (RT-PCR) to amplify 244 bp of the matrix segment of AIV, according to World Health Organization (WHO) protocol (*12*). Samples with amplified M segments were subjected to H5, H7, and H9 hemagglutinin subtype determination by RT-PCR according to the same WHO protocol, except the annealing temperature for H5 primers was 58°C (*12*,*13*). As of August 2012, quantitative RT-PCR (qRT-PCR) was used for typing and subtyping AIV. Typing and subtyping primers are listed in Table 1.

**Table 1:** Primers used for H5 and H9 subtyping of avian influenza viruses from Egypt, 2010–2012*

Primer	Sequence, 5′→3′	Reference
M30F2/08	ATGAGYCTTYTAACCGAGGTCGAAACG	(*12*)
M264R3/08	TGGACAAANCGTCTACGCTGCAG	
H5–155f	ACACATGCYCARGACATACT	(*13*)
H5–699r	CTYTGRTTYAGTGTTGATGT	
H9–151f	CTYCACACAGARCACAATGG	
H9–638r	GTCACACTTGTTGTTGTRTC	
BDH9–4F2	CAAGCGTGACAACAGAAAATTTGG	Designed in-house†
BDH9–2R2	CTCCTGAGAGAACGTGTCCATACC	
H9PROB	FAM CTTACTCGCAATGTCTGGCCTGGTTTTAG BHQ1	
AH5b_Forward	GGA ATGYCCCAAATATGTGAAATCAA	(*14*)
AH5b_R	CCACTCCCCTGCTCRTTGCT	
H5PROB	FAM TACCCATACCAACCATCTACCATTCCC BHQ1	
Inf-A F	ACCRATCCTGTCACCTCTGAC	
Inf-A R	AGGGCATTYTGGACAAAKCGTCTA	
Inf-A POB	FAM TGCAGTCCTCGCTCACTGGGCACG BHQ1	

*Typing by reverse transcription PCR and quantitative reverse transcription PCR. †St. Jude Children’s Research Hospital, Memphis, TN, USA.

### Virus Isolation

Samples that showed a positive reaction in the partial M segment RT-PCR were grown in the allantoic cavities of 10-day-old specific pathogen–free embryonated chicken eggs. Virus titers were determined by chicken red blood cell hemagglutination assays (*12*).

### Hemagglutinin Gene Sequencing and Sequence Analyses

The purified amplicons of 26 H5 and 15 H9 segments, selected to represent time and species, were sequenced as described (*15*). Phylogenetic analyses were performed by using MEGA version 4.0.2 (www.megasoftware.net) with the neighbor-joining method and Poisson correction (*16*). The sequences were submitted to GenBank under accession nos. KF258174–91, CY099582–8, CY099591–3, JX912982–6, JX912988, JX912990–2, and JX912994–7.

### Antigenic Cartography

Chicken red blood cell hemagglutination inhibition (HI) assays were conducted in accordance with the WHO protocol (*12*) and with monoclonal antibodies against influenza virus subtype H5N1. Antigenic maps were constructed by using virus titers and AntigenMap software (*17*).

### Influenza Virus Subtype H5N1 and H9N2 Co-infection

Because co-infection with influenza virus subtypes H5N1 and H9N2 can give way to viral reassortment and production of viral progeny with unpredictable phenotypes, we studied co-infected samples in more detail. The presence of the 2 viruses in 3 selected samples collected in 2012 (Q5018B, D5809C, D5809D) was detected by RT-PCR, qRT-PCR, hemagglutinin sequence analysis, immunofluorescence, and Western blotting. The samples were propagated in specific pathogen–free embryonated chicken eggs, and the allantoic fluid was subjected to qRT-PCR. To separate the 2 viruses, we then conducted a plaque purification assay. Individual plaques were picked and injected into specific pathogen–free embryonated chicken eggs and MDCK cells (in the presence or absence of L-1-tosylamide-2-phenylethyl chloromethyl ketone [TPCK]–treated trypsin). After incubation, the allantoic fluids and cell culture supernatant were harvested and subjected to qRT-PCR.

### Plaque Purification

A 100-μL sample of the original decontaminated samples and 10-fold serial dilutions from each sample were inoculated into wells of a 6-well plate containing confluent MDCK cells with 400 μL serum-free medium. The plates were incubated at 37°C for 1 hour. The wells were aspirated to remove residual viral solution. Each well was then immediately covered with 2 mL 1× agarose overlay mixture (final concentration 1% agarose type 1, 1× Dulbecco modified Eagle medium, 10% antibiotic/antimycotic solution). Plates were then incubated at 37°C under 5% CO_2_ for 2 days. Plaques were picked, and each plaque was inoculated into specific pathogen–free embryonated chicken eggs for propagation of purified plaques.

### Immunofluorescence

MDCK cells were inoculated with original co-infected specimens. At 1 day after inoculation, the cells were fixed with 1 mL 3.7% formalin in PBS for 5 min, and then 1 mL cold methanol was added for 5 min. Cells were blocked by using 1 mL 1% bovine serum albumin (Serva, Heidelberg, Germany) in PBS-Tween 20 at 37°C for 1 hour. Rat and chicken antiserum against H5N1 and H9N2 viruses, respectively, were incubated with the fixed cells. Fluorescein isothiocyanate–conjugated goat anti–chicken IgG and goat anti–rat IgG diluted 1:2000 (KPL, Gaithersburg, MD, USA) were then added. Fluorescently labeled cells were examined by using fluorescence microscopy.

### Western Blotting

Viruses propagated in specific pathogen–free embryonated chicken eggs were analyzed by SDS PAGE (sodium dodecyl sulfate polyacrylamide gel electrophoresis) as described (*18*); the only modification was that 1% bovine serum albumin in PBS–0.3% Tween20 was used to block the protein-free binding sites on the nitrocellulose membrane. Immunorecognition was performed on cut membrane strips carrying chicken anti-H9N2 serum (dilution 1:50) or mouse anti-H5 monoclonal antibody (dilution 1:100). Immune detection was conducted by using peroxidase-conjugated goat anti–chicken IgG and goat anti–mouse IgG (KPL) diluted 1:2000 in PBS–0.3% Tween20.

### Propagation Rates

Equal titers of influenza virus subtypes H5N1 and H9N2 were separately or jointly inoculated into specific pathogen–free embryonated chicken eggs and MDCK cells. The amount of virus propagated was measured by qRT-PCR at 24 hours after inoculation.

### Statistical Analysis

The proportion of samples that were positive according to the different study variables was determined. The Pearson χ^2^ test was used

## Results

### AIV in Poultry

Test results were positive for 1,144 birds (Table 2); the overall percentage of AIV detected was 10% (95% CI 9.5%–10.5%). This percentage differed significantly according to governorate, species, production sector, health status, and age (Table 2). The detection percentage by governorate ranged from 3% in Menofiya to 18% in Daqahliya (Figure 1). Detection percentage for urban Cairo was 6.4%. Of the swab samples collected, 84.2% were from chickens, 10.3% were from ducks, and 5.5% were from other species of domestic birds (Table 2). The detection percentage was highest among turkeys (15.3%); followed by ducks (12.3%); and then chickens, pigeons, and geese (≈9%). Among swab samples collected from the different poultry sectors, the highest percentage positive for AIV (≈12%) came from commercial farms and backyard flocks, followed by live-bird markets (6.7%) and abattoirs (5.1%). Most (88.3%) swab samples were collected from apparently healthy birds; of those, 8.3% were positive for AIV. Influenza A viruses were detected in 20.8% of sick and 42.4% of dead birds. The detection percentage among poultry >1 year of age was 19.4%; that among birds <1 year of age was 9.9%.

**Table 2:** Epizootiologic data for avian influenza virus isolated from poultry in 7 governorates in Egypt, 2010–2012

Variable	Samples collected, no. (%)	Influenza A–positive samples, no. (%)	p value
Sample type			Not significant
Cloacal	6,904 (60.3)	686 (9.9)	
Oropharyngeal	4,548 (39.7)	458 (10.1)	
Governorate			<0.001
Cairo	2,690 (23.4)	173 (6.4)	
Daqahliya	1,440 (12.6)	259 (18)	
Qalubiya	1,478 (12.9)	141 (9.5)	
Menofiya	935 (8.2)	28 (3)	
Sharqiya	2,365 (20.7)	236 (10)	
Fayyoum	2,006 (17.5)	262 (13.1)	
BeniSuef	538 (4.7)	45 (8.4)	
Species			0.023
Chickens	9,639 (84.2)	938 (9.7)	
Ducks	1,179 (10.3)	145 (12.3)	
Geese	139 (1.2)	12 (8.6)	
Pigeons	410 (3.6)	36 (8.8)	
Turkeys	85 (0.7)	13 (15.3)	
Production sector			<0.001
Abattoir	992 (8.7)	51 (5.1)	
Commercial farm	6,398 (55.8)	745 (11.6)	
Backyard flock	1,261 (11)	159 (12.6)	
Live-bird market	2,801 (24.5)	189 (6.7)	
Bird health status			<0.001
Healthy	10,117 (88.4)	841 (8.3)	
Sick	1,217 (10.6)	35 (20.8)	
Dead	118 (1)	50 (42.4)	
Bird age, y			<0.001
0–1	11,328 (98.9)	1120 (9.9)	
>1	124 (1.1)	24 (19.4)	

**Figure 2:**
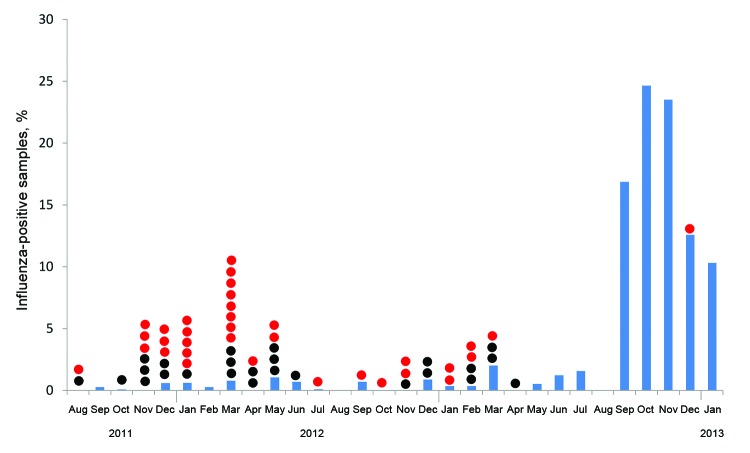
Avian influenza virus infections, by month, Egypt, 2011–2013. Blue bars, detection of the virus in birds; red dots, cases of influenza A(H5N1) virus infections in humans; and black dots, human deaths from influenza A(H5N1) virus infection.

More AIV was detected in poultry during colder months, and none was detected in August (Figure 2; Technical Appendix Figure). Among poultry, the detection percentage for AIV was <5% until September 2012, when we recorded a sudden increase of >15%. This outbreak peaked at ≈25% in October 2012 and continued into January 2013 (Figure 2). This outbreak was detected at all of our sampling sites and was more pronounced in Cairo, Fayyoum, and BeniSuef, where we did not detect AIV before this outbreak (Technical Appendix Figure 1).

**Figure 3:**
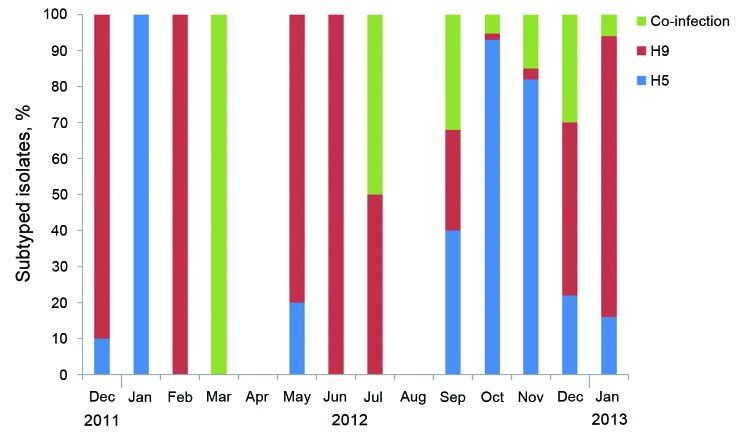
Subtypes of influenza A viruses detected in poultry, by month, by using reverse transcription PCR, Egypt, 2011–2013.

Of the 1,144 influenza virus–positive samples, we subtyped 897. From August 2010 through November 2011, all 59 AIV samples were subtype H5N1. Figure 3 shows the percentage of each subtype identified from December 2011 through January 2013. In December 2011, we detected the first subtype H9N2 virus in our surveillance program. In March 2012, we detected the first subtype H5N1 and H9N2 co-infections. We then detected 151 incidences of co-infection throughout the reporting period. During the September 2012–January 2013 outbreak, detection of AIV increased dramatically; thus, we randomly selected positive samples from that period for subtyping. During September–November 2012, subtype H5N1 and co-infections constituted most (92%) subtypes detected. In December 2012, detection of subtype H5N1 decreased. Overall, subtype H5N1 was the dominant subtype by governorate, species, production sector, health status, and age (Table 3). Throughout this period, we isolated 112 viruses in specific pathogen–free embryonated chicken eggs.

**Table 3:** Epizootiologic data for avian influenza virus subtypes H5N1, H9N2, and H5/H9 , Egypt, 2010–2012

Variable	No.	H5N1, %	H9N2, %	H5/H9, %
Governorate				
Cairo	132	99.2	0.8	0
Daqahliya	175	50.9	17.7	31.4
Qalubiya	92	71.7	5.4	22.8
Menofiya	28	96.4	3.6	0
Sharqiya	200	54.5	28.0	17.5
Fayyoum	225	72	13.8	14.2
BeniSuef	45	82.2	0	17.8
Species				
Chickens	709	62.9	17.5	19.6
Ducks	139	90.6	0.7	8.6
Geese	12	100	0	0
Pigeons	25	100	0	0
Turkeys	12	100	0	0
Production sector				
Abattoir	42	100	0	0
Commercial farm	572	59.6	18.7	21.7
Backyard flock	145	85.5	7.6	6.9
Live-bird market	138	82.6	5.1	12.3
Bird health status				
Healthy	660	66.8	14.2	18.9
Sick	147	75	15.8	9.2
Dead	41	80.5	0	19.5
Bird age, y				
0–1	608	69.6	14.3	16
>1	13	54.2	0	45.8

### Phylogenetics

**Figure 4:**
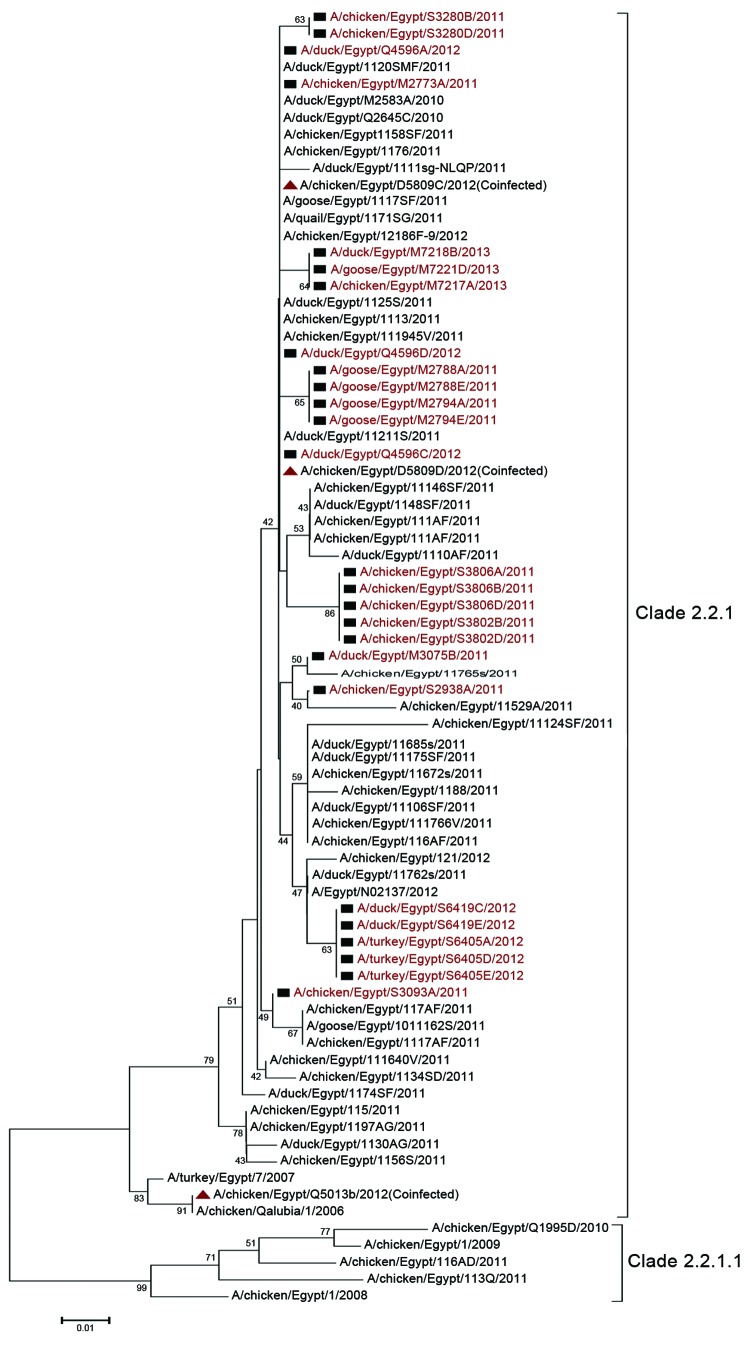
Phylogenetic tree of the hemagglutinin gene of influenza A(H5N1) viruses from Egypt, 2010–2012. Squares and red text indicate viruses that were isolated and sequenced as part of the study. Black text indicates sequences available on GenBank from previous years or other groups. Triangles indicate co-infection with influenza virus subtypes H5N1 and H9N2. Scale bar indicates phylogenetic distance (1 base substitution/100 positions).

We constructed a phylogenetic tree of the hemagglutinin gene of influenza A(H5N1) viruses from Egypt (Figure 4). Clade 2.2 viruses circulated during 2006–2007 and were distinct from those that prevailed during 2008–2009, when the virus was declared enzootic. Clades 2.2.1 and 2.2.1.1 viruses cocirculated from 2010 through mid-2011. Clade 2.2.1.1 then receded, and all viruses isolated from late 2011 through 2013 were from clade 2.2.1. The subtype H5N1 virus sequence obtained from samples that were co-infected with subtype H9N2 virus did not differ from the sequences of clade 2.2.1 viruses. However, 1 subtype H5N1 virus from a co-infected sample (A/chicken/Egypt/Q5013B/2012) clustered with the extinct clade 2.2 viruses. No other virus from recent years has had a similar sequence.

**Figure 5:**
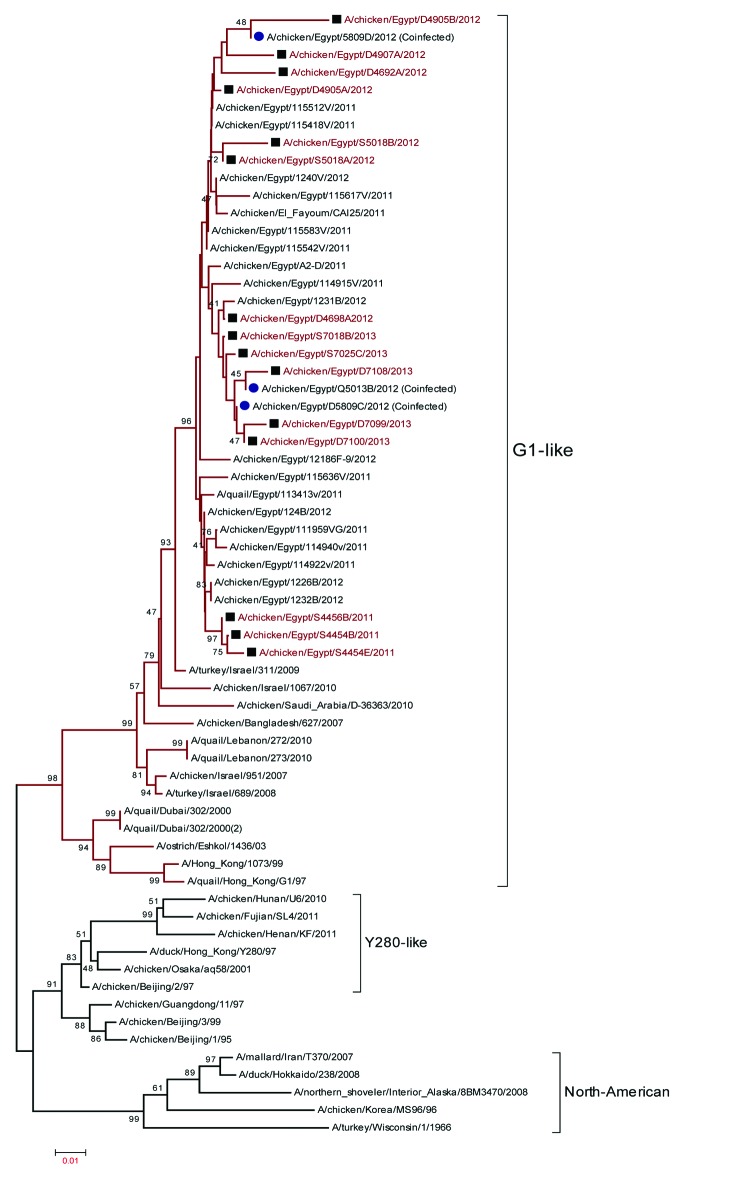
Phylogenetic tree of the hemagglutinin gene of influenza A(H9N2) viruses from Egypt, 2010–2012. Squares and red text indicate viruses that were isolated and sequenced as part of the study. Black text indicates sequences available on GenBank from previous years or other groups. Circles indicate co-infection with influenza virus subtypes H5N1 and H9N2. Scale bar indicates phylogenetic distance (1 base substitution/100 positions).

Phylogenetic analysis of the hemagglutinin gene of subtype H9N2 viruses that reemerged in Egypt in 2011 indicated that only viruses with a G1-like lineage circulated among poultry in Egypt (Figure 5). Influenza A(H9N2) viruses from Egypt clustered together, thus showing minor evolution during the past 2 years. Sequences obtained from samples co-infected with subtype H5N1 also showed no significant differences from the sequences of subtype H9N2 viruses that were not from co-infected samples.

### Antigenic Characterization of Influenza A(H5N1) Viruses

Results of HI assay of the 2012–2013 viruses conducted against a panel of monoclonal antibodies were used to update a previously published antigenic cartograph (*3*). Our results indicate that antigenically, subtype H5N1 viruses from Egypt have drifted over time; in 2010, two clusters of viruses (clades 2.2.1 and 2.2.1.1) cocirculated. In 2011–2013, clade 2.2.1 viruses dominated (Technical Appendix Figure 2).

### Clinical Signs, 2006–2013

We previously reported our field observations of illness and death caused by HPAI viruses in poultry in Egypt (*19*). Briefly, during 2006–2007, when clade 2.2 viruses were circulating, mortality rates were up to 100%. During 2008–2009, when the virus became enzootic, mortality rates dropped to 30%–40%. Here we show that in 2010, when viruses from clades 2.2.1 and 2.2.1.1 cocirculated, mortality rates were 20%–60%, and this trend continued into 2011. Subsequently, the commercial farms we surveyed decreased their vaccine use. By 2012, as clade 2.2.1.1 viruses receded, mortality rates started to increase. Samples that indicated co-infection with subtypes H5N1 and H9N2 came from flocks that were killed because of the infection. Furthermore, the September 2012 outbreak was caused by viruses that caused high mortality rates among commercial and backyard flocks. In fact, most (96%) influenza-positive samples that were collected from sick or dead poultry were collected during this outbreak.

### Co-infections 

The presence of influenza virus subtypes H5N1 and H9N2 in the 3 selected samples was confirmed by RT-PCR, qRT-PCR, hemagglutinin sequence analysis, immunofluorescence, and Western blotting (Technical Appendix Figure 3). In egg culture, subtype H9 virus grew faster than did subtype H5 after 1 passage in eggs, although the cycle thresholds for both viruses were the same for the original swab sample (Technical Appendix Figure 4). To separate the 2 viruses, we then conducted a plaque purification assay. All plaques that were individually picked from the plaque assay and propagated on egg or cell culture were subtype H9N2 viruses.

To understand our inability to isolate subtype H5N1 virus by plaque purification, we conducted an experimental co-infection analysis of both viruses at an equal dose of 100 PFU/mL in MDCK cells and in specific pathogen–free embryonated chicken eggs. Our results showed that 24 hours after inoculation, both viruses grew to similar titers in MDCK cells in the presence of TPCK-treated trypsin, and the presence of the other virus in the culture did not affect propagation (Technical Appendix Figure 5). In eggs, subtype H9N2 virus grew more efficiently than did subtype H5N1 virus, but the presence of the other virus did not affect the propagation rate of either virus (Technical Appendix Figure 5).

## Discussion

AIV subtypes H5N1 and H9N2 were very common among domestic poultry in Egypt. The highest percentage of AIV detected was among turkeys and ducks that appeared to be healthy. Ducks in Egypt, like those in other regions, play a key role in AIV transmission (*20*).We also detected AIV in chickens in all sectors of production. Virus detection among pigeons sampled at markets and abattoirs was 8.8%, but no viruses were isolated from these birds. Therefore, pigeons might have become incidental carriers while coming in contact with other infected poultry in live-bird markets at which all the pigeons included in our surveillance were swabbed. Percentage of AIV detection was high (≈12%) at commercial farms and backyard flocks, where chickens, ducks, geese, and turkeys were sampled. In our previous analysis of surveillance data obtained from August 2009 through July 2010, the commercial farm sector was a more common reservoir of AIV than was the backyard sector (*11*). The continuous evolution of the virus and the disappearance of clade 2.2.1.1 viruses that predominantly circulated on commercial farms might explain the findings in our current analysis. AIV detection rates at abattoirs in Cairo and live-bird markets at different sites were ≈6%. This finding suggests that the threat of bird-to-human transmission might extend beyond the backyard setting, where most cases of subtype H5 infection in humans in Egypt were reported. Our previous analysis had the same result (*11*). Influenza virus infection in apparently health poultry increased from 4.5% in the previous period to 8.3%. This finding might be caused by subtype H9N2 virus infections that are mainly asymptomatic in poultry.

During our surveillance period, influenza A(H5N1) virus infections among humans occurred in a seasonal pattern that peaked during February and March (Figure 2). These cases in humans occurred mostly during months when influenza activity was detected in poultry. Globally, most humans with influenza A(H5N1) virus infection reported having had contact with sick or dead poultry (*21*). The decrease in the number of cases in humans in late 2012, when infections in poultry increased, remains unexplained, although this decrease occurred at a time when human cases usually peak. The political situation during this period might have affected case detection and reporting.

Previous studies have documented the presence of other influenza virus subtypes in migratory birds in Egypt, although none have reported isolating those subtypes from domestic poultry (*22*–*24*). In contrast, subtype H9N2 viruses have been detected in domestic poultry in several neighboring Middle Eastern countries (*25*–*29*). As of May 2011, subtype H9N2 viruses were detected on quail and chicken farms in Egypt by another group (*10*), and in December 2011, they were detected by our surveillance. However, how influenza virus subtype H9N2 was introduced into Egypt remains unclear.

Using RT-PCR, we detected a substantial rate of co-infection with influenza virus subtypes H9N2 and H5N1. This cocirculation and co-infection of multiple influenza viruses increases the chances of subtype H5N1 virus reassortment. Our phylogenetic and antigenic analyses of subtype H5N1 viruses indicated that clade 2.2.1.1 viruses receded and that 2.2.1 viruses are widely circulating. The emergence and recession of clade 2.2.1.1 viruses warrants further investigation of the role that vaccine use plays in emergence of variants. During September 2012–January 2013, increased detection (as much as 24%) of clade 2.2.1 virus infection was noticed. These viruses were associated with high mortality rates and were responsible for the September 2012 outbreak. Furthermore, this shift from the <5% that was detected before that time was substantial. Subtype H9N2 viruses might have played a role in this increased spread and severity through an unknown mechanism. However, subtype H9N2 virus did not mask infection with subtype H5N1 virus, as previously suggested (*30*). Yet in co-infected samples, subtype H9N2 might be more easily detected, especially if culture or serologic assays were used, because our data indicate that subtype H9N2 virus grows faster than subtype H5N1 virus in co-infected field samples. Subtype H9N2 virus was circulating in neighboring Middle Eastern countries since at least 2000 but was not detected in Egypt until May 2011; therefore, we hypothesized that subtype H9N2 virus emerged when the pathogenicity of subtype H5N1 virus decreased. Accordingly, we repeated our co-infection experiments with subtype H5N1 viruses isolated annually during 2006–2011. The results were similar to the subtype H9N2/H5N1 virus co-infections in 2012, showing that varying subtype H5N1 virus pathogenicity over time was not a factor in the emergence of subtype H9N2 virus in Egypt (data not shown). Factors that led to the emergence of subtype H9N2 virus and the consequences of its co-infection with subtype H5N1 virus remain unclear.

Through our systematic surveillance program of AIV in poultry in Egypt, we were able to detect 3 major events: emergence of subtype H9N2 virus, co-infection of single hosts with subtypes H9N2 and H5N1 viruses, and increased detection of AIV as of September 2012. We determined that the reservoir for AIV is not localized to a specific sector of poultry production in Egypt, is not specific to a single species, and is geographically widespread. Although our findings indicate that cocirculation and co-infection with AIVs are low, these events are of major concern because of their high potential for reassortment, which can lead to virus progeny with novel characteristics that threaten not only avian health but also human health.

Our study had several limitations. First, our surveillance did not include all geographic areas, and southern Egypt was not adequately represented. The climate in southern Egypt differs from that in the Delta, and the presence of the Aswan Dam and Nasser Reservoir might affect the epizootiology of influenza viruses given the presence of wild bird species. Furthermore, we did not sample any wild or migratory birds, thus limiting our findings, especially those associated with the emergence of subtype H9N2 virus. In areas where we conducted our surveillance, we did not randomly select sites; rather, we sampled sites that were accessible to our veterinarians. Although the possibility is small, given our sampling scheme, selection bias might have occurred.

Our findings showed that influenza viruses continue to threaten animal and human health in Egypt. In the poultry industry, HPAI A(H5N1) viruses usually lead to major economic losses. Subtype H9N2 viruses, although of low pathogenicity, are correlated with increased severity because of co-infection with other poultry viruses; thus, they indirectly might lead to economic losses for the industry. On the public health side, our findings that AIVs are widespread throughout poultry sectors and geographic regions indicate that a large segment of the population of Egypt is at risk. Subtype H9N2 viruses also infect humans, thereby adding to the risk for infection with subtype H5N1 virus. Our results can be used to better focus and target animal health and public health policy in Egypt. Indeed, Egypt remains an epicenter for AIV circulation, and vigilant surveillance remains the single-most effective tool for keeping track of these viruses.

Technical Appendix Avian influenza A virus detection in poultry in Egypt, by month and by subtype; experimental growth of avian influenza A virus in specific pathogen–free embryonated chicken eggs and MDCK cells; and antigenic cartograph of influenza A(H5N1) viruses isolated during 1997–2013.
