# Supplementary material for: Active Surveillance for Avian Influenza Virus, Egypt, 2010–2012
Source: Emerg Infect Dis. 2014 Apr;20(4):542–51. doi: 10.3201/eid2004.131295 (PMC3966394; doi:10.3201/eid2004.131295)
Supplement: Technical Appendix — Avian influenza A virus detection in poultry in Egypt, by month and by subtype; experimental growth of avian influenza A virus in specific pathogen–free embryonated chicken eggs and MDCK cells; and antigenic cartograph of influenza A(H5N1) viruses isolated during 1997–2013. [file 13-1295-Techapp-s1.pdf]

Article DOI: http://dx.doi.org/10.3201/eid2004.131295

Active Surveillance for Avian Influenza Virus, Egypt, 2010–2012

Technical Appendix

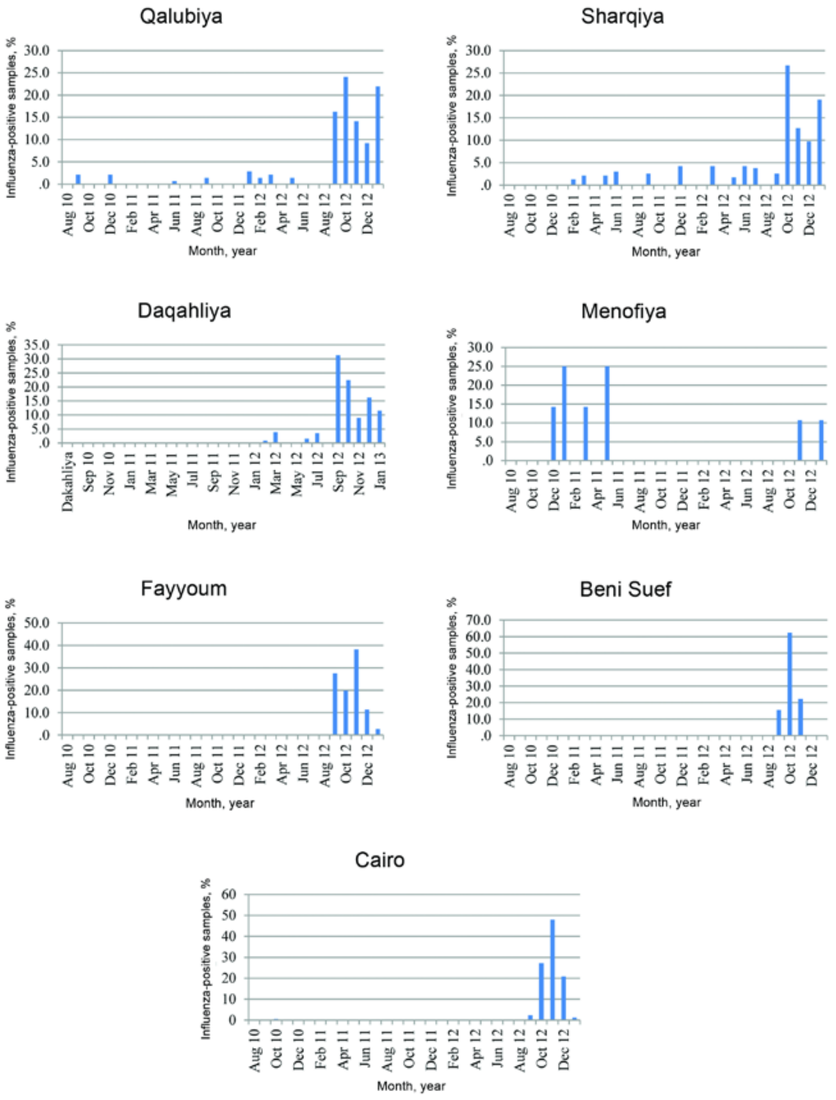

Technical Appendix Figure 1. Detection of influenza A viruses in poultry in Egypt by using reverse transcription PCR. Surveillance results for August 2010–January 2013 are presented by month. Separate plots indicate results from individual governorates.

Figure 6

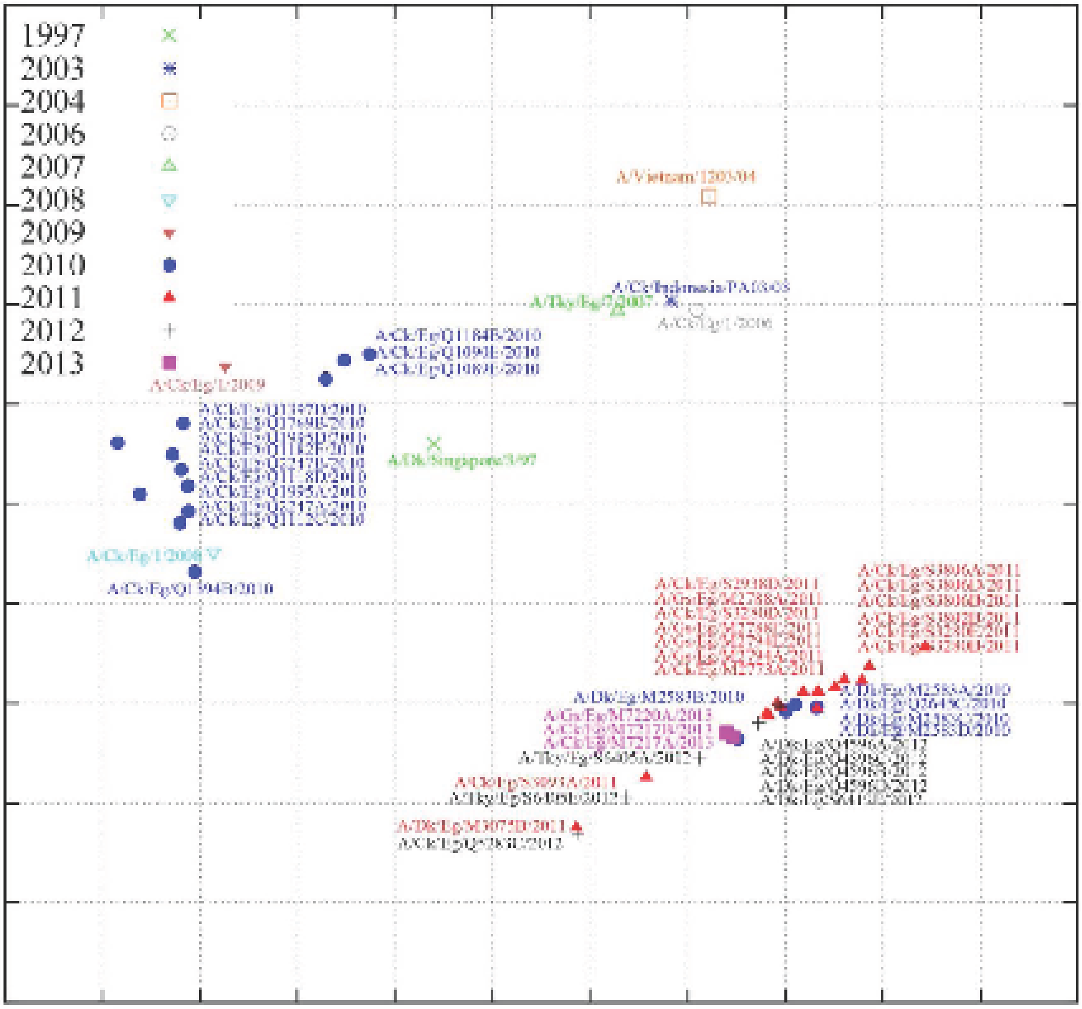

.

Technical Appendix Figure 2. Antigenic cartograph of influenza A(H5N1) viruses isolated during 1997–2013, generated by using hemagglutination-inhibition assay data and AntigenMap software (*17*). Each square on the grid represents a 2-fold change in the assay result. This figure represents an update of a previously published map (*6*).

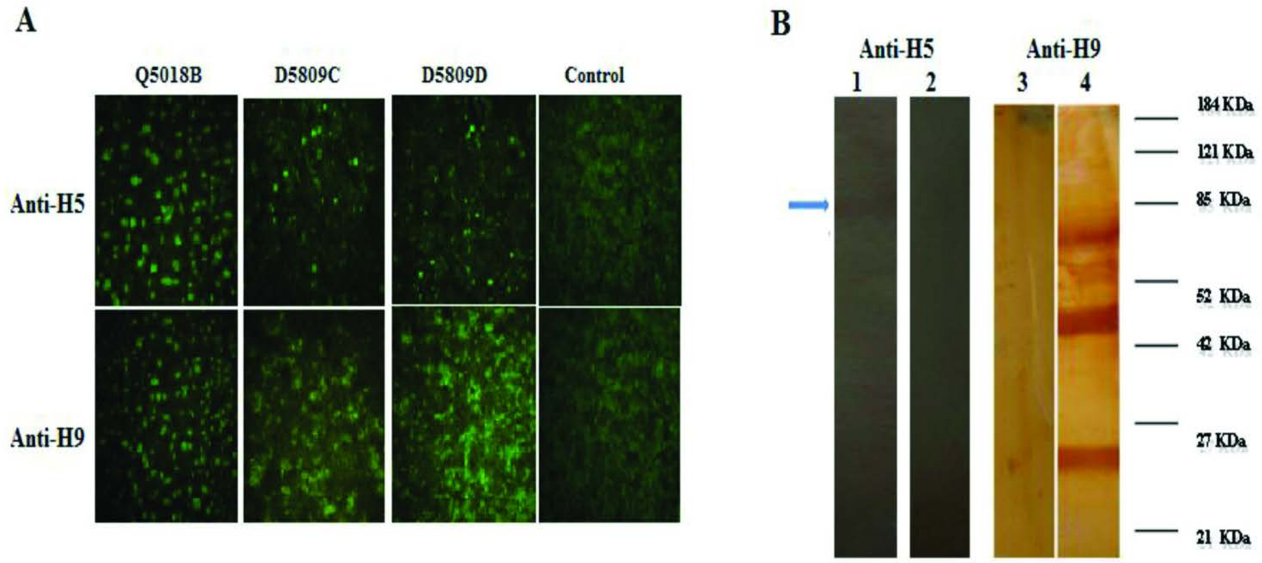

Technical Appendix Figure 3. Influenza A virus subtypes H5 and H9 co-infection in single samples from poultry, Egypt, 2010–2012. A) Immunofluorescence with rat anti-H5 and chicken anti-H9 serum shows co-infection with subtypes H5 and H9 virus in 3 samples (Q5013B, Q5809C, and D5809D). B) Western blot analysis of sample Q5018B with a monoclonal antibody against H5 and chicken anti-H9 serum. The blue arrow indicates a band on the subtype H5 Western blot.

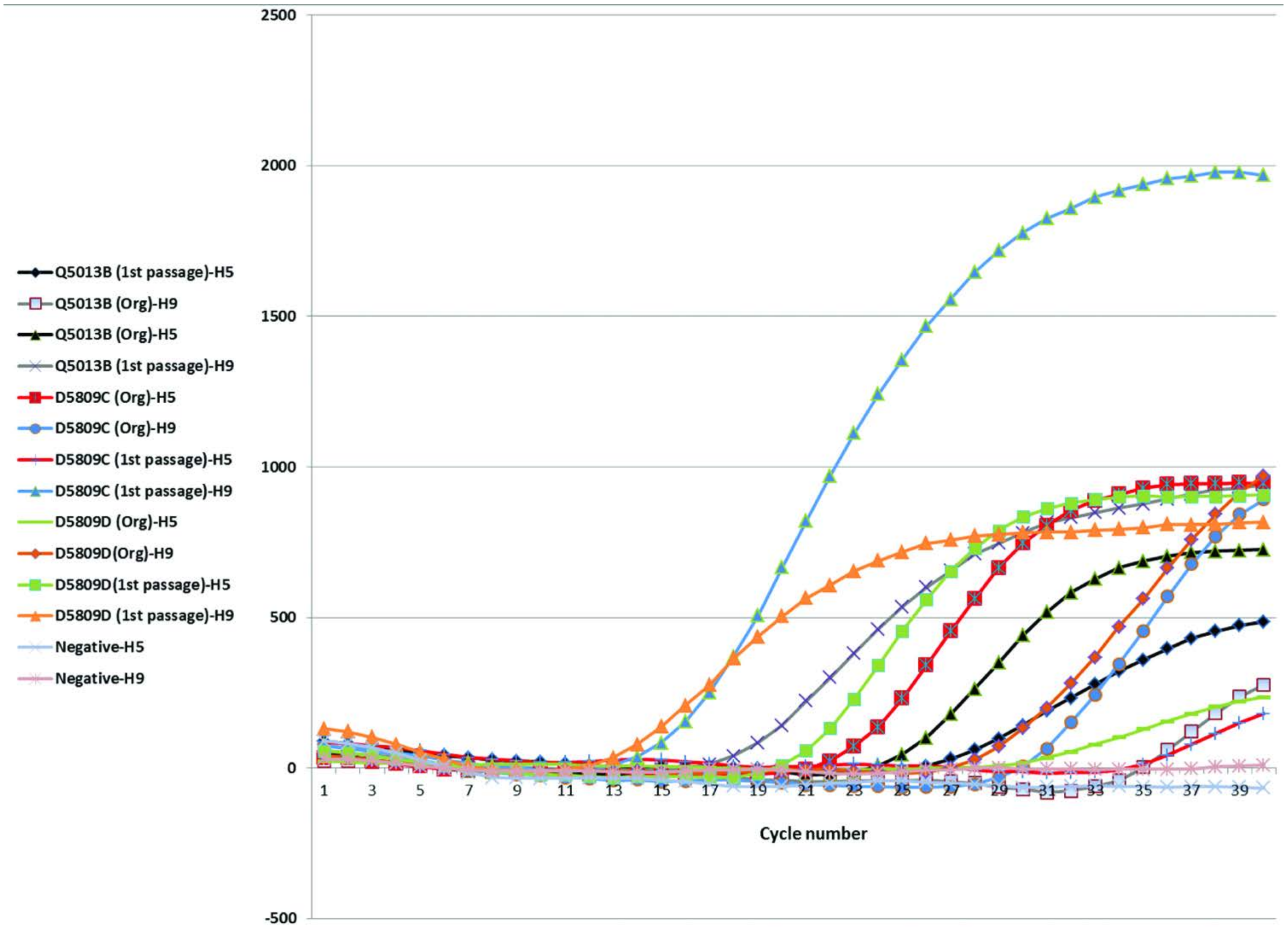

Technical Appendix Figure 4. In embryonated chicken egg, influenza A virus subtype H9 grows faster than subtype H5, as determined by quantitative reverse transcription PCR analysis. Org, original swab sample from the bird.

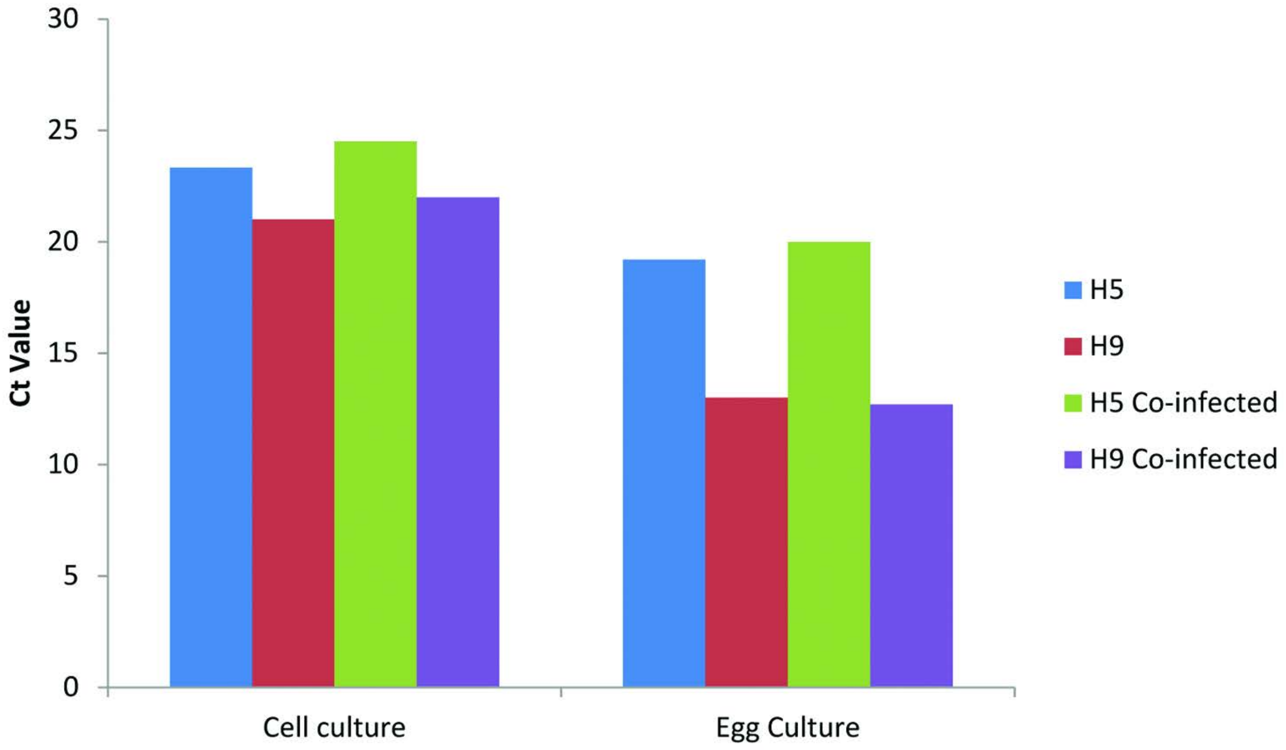

Technical Appendix Figure 5. Experimental co-infection of influenza virus subtypes H5N1 and H9N2 in specific pathogen–free embryonated chicken eggs and MDCK cells did not affect the propagation of either virus. Virus titers in eggs and cells infected with virus subtype H5N1 (blue bars) or H9N2 (red bars) virus alone and those co-infected with both viruses (green and purple bars) did not differ significantly.
